# The clinical significance of microvascular invasion in the surgical planning and postoperative sequential treatment in hepatocellular carcinoma

**DOI:** 10.1038/s41598-021-82058-x

**Published:** 2021-01-28

**Authors:** Wentao Wang, Yaxun Guo, Jingtao Zhong, Qi Wang, Xin Wang, Honglong Wei, Jie Li, Peng Xiu

**Affiliations:** 1 Department of General Surgery, Shandong Qianfoshan Hospital, Cheeloo College of Medicine, Shandong University, Jinan, 250014 Shandong China; 2 Department of General Surgery, Shandong Provincial Qianfoshan Hospital, the First Hospital Affiliated With Shandong First Medical University, Jinan, China; 3 Department of Hepatobiliary Surgery, Shandong Cancer Hospital and Institute, Shandong First Medical University and Shandong Academy of Medical Sciences, Jinan, China

**Keywords:** Cancer, Microbiology

## Abstract

Hepatocellular carcinoma (HCC) is one of the most common and most lethal malignant tumors in the world. Microvascular invasion (MVI) is a major risk factor for survival outcomes and intrahepatic metastasis after resection in patients with HCC. Relevant English literatures retrieved using PubMed on the research progress of MVI in patients with HCC were reviewed. For HCC patients, especially those with MVI, it is very important to develop a comprehensive and sequential treatment plan to support the long-term survival of patients. This manuscript reviewed and analyzed the risk factors for MVI; the preoperative prediction of MVI, which informs the selection of surgical strategies; and the current situation and future direction of comprehensive postoperative treatment strategies; to provide a basis for the comprehensive treatment of HCC patients with MVI. For HCC patients with MVI, the preoperative prediction of MVI may play a certain guiding role in planning procedures, and the comprehensive sequential postoperative pathological detection of HCC MVI may provide a basis for treatment decisions.

## Introduction

HCC is the fifth most common cancer worldwide^1^ and the third most common cause of cancer-related death, with more than half of the new cases and deaths occurring in China every year^2^. In the past two decades, substantial progress has been made in the treatment of HCC with advances in medical technology, although surgical resection and liver transplantation are still the most effective treatments. However, the prognosis of patients with HCC after surgical resection is very poor. A high recurrence rate and a high mortality rate are important factors affecting the long-term survival status of patients after surgical treatment. The 5-year recurrence rates after surgical treatment and liver transplantation are as high as 70% and 35%, respectively^3^. Current research advances suggest that there are many risk factors for tumor recurrence after surgical resection, including residual lesions, vascular invasion, HBV/HCV replication, degree of liver cirrhosis, the presence and the degree of portal hypertension (HP) and surgical methods^4^. Among them, vascular invasion is considered a key factor in the early recurrence (within 24 months of surgery) of HCC based on the results of multiple retrospective studies^3,5,6^. Therefore, it is very clinically important to explore the selection of treatment strategies after the surgical resection of HCC, especially in patients with vascular invasion.

Vascular invasion is considered either macroscopic or microscopic. The former is determined by gross tissue evaluation or diagnostic imaging, whereas the latter, microvascular invasion (MVI), is determined by a histopathological examination of the tumor and surrounding hepatic tissue^7^. Although multiple recent retrospective analyses have found close correlations between MVI and adverse tumor biology and a poor prognosis, the choice of the appropriate treatment for HCC patients complicated with MVI is still worthy of an in-depth discussion.

## Definition of MVI

**Figure 1:**
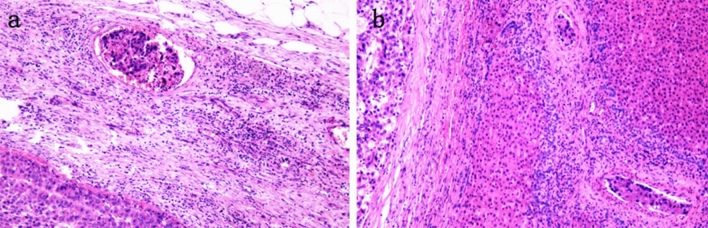
Peripheral vascular invasion of HCC tissues. MVI appears as nests of malignant cells lining the vascular cavities of endothelial cells or portal and hepatic venous systems. (**a**) The presence of tumor cells in a vein. (**b**) The presence of tumor cells in small arteries.

**Figure 2:**
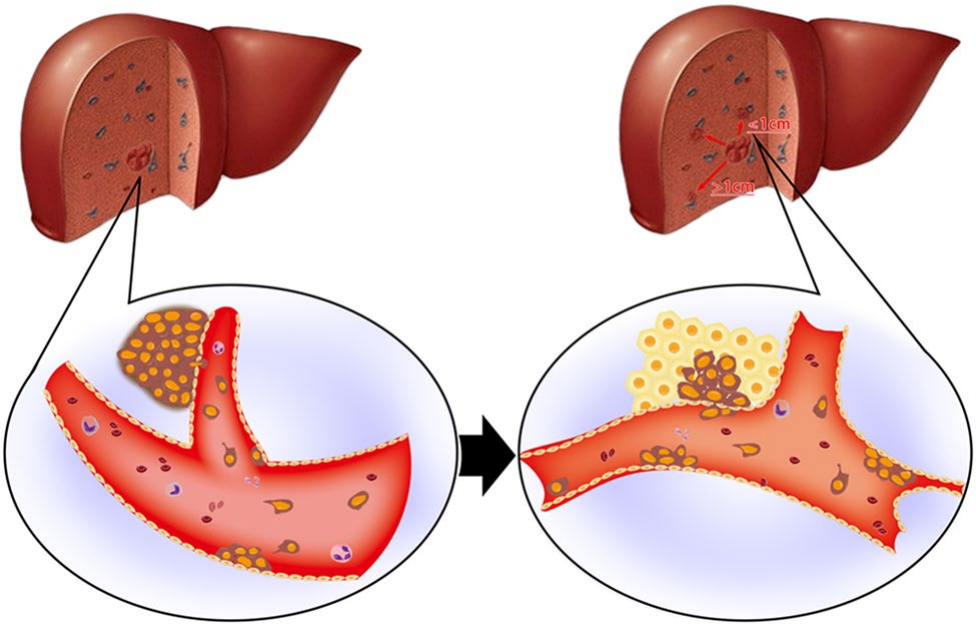
MVI classification and its presence in HCC. MVI be evaluated in all tissue sections and graded according to the level of risk based on the number and distribution of sites as follows: M0, no MVI; M1 (low-risk), MVI < 5 and at ≤ 1 cm away from the adjacent liver tissue; and M2 (high-risk), MVI > 5 or at > 1 cm away from the adjacent liver tissue.

Strictly speaking, MVI is a pathological diagnosis and there are many definitions and standards for MVI. Microscopically, MVI appears as nests of malignant cells lining the vascular cavities of endothelial cells or portal and hepatic venous systems (Fig. 1)^8^. MVI can be evidenced by the identification of tumor cells within endothelial-lined spaces on standard hematoxylin and eosin stained slides^7^. Previous studies have shown that the incidence of MVI fluctuates between 15 and 57.1% in HCC patients^9^. This difference may be related to the sampling site and the non-uniform diagnostic criteria. Cong et al. drew conclusions from previous research results, and they recommend that MVI be evaluated in all tissue sections and graded according to the level of risk based on the number and distribution of sites as follows: M0, no MVI; M1 (low-risk), MVI < 5 and at ≤ 1 cm away from the adjacent liver tissue; and M2 (high-risk), MVI > 5 or at > 1 cm away from the adjacent liver tissue (Fig. 2)^10^. This was also the first detailed description of the definition of MVI in a pathology guide in China.

Hepatocyte canceration usually occurs on the basis of chronic inflammatory stimulation, biological factors, toxic exposure and metabolic abnormalities, activating proto-oncogenes, inactivating tumor suppressor genes through multiple signaling pathways, leading to inhibition of cell apoptosis and triggering uncontrolled cell proliferation. Tumor cells can secrete cathepsin H to break down extracellular matrix, and at the same time reduce the synthesis of cadherin E to reduce the adhesion between cells, so as to facilitate tumor metastasis. Tumor-induced neoangiogenesis, proliferation and inhibition of apoptosis are the prime reasons of MVI^11^. Among them, degradation of the basal membrane and herniation of the tumor cells to the capillary lumen is the key step in the process of MVI. In this process, circulating tumor cells are coated with endothelial cells and form microemboli in the capillary lumen. When MVI occurs, it indicates that HCC cells have invaded blood vessels and may begin to metastasize, and the tumor has progressed to a new stage. Small blood vessels in the liver are composed of vascular endothelial cells, fibronectin and fibrinogen basement membrane. The cluster of hepatocellular carcinoma cells can be embedded in the vascular endothelial cells and surrounded by the vascular endothelial cells to survive in the blood, so as to escape the immune cell clearance of the body and avoid activating the coagulation mechanism to achieve distant metastasis^12^.

## Risk factors associated with MVI

### Tumor classification (Table 1)

**Table 1:** Risk factors associated with MVI.

Author	Year	No	Treatment received	Risk factors
He et al^16^	2015	144	Resection	Type IV, infiltrative type (MVI rate: 63.5%; OS: 45.9 ± 5.1 months, *P* < 0.05)
Cho et al^17^	2015	Not stated	Resection	Tumors with more than 3 foci Tumor size > 3 cm Multiple tumors or tumors > 3 cm with non-smooth margins on MRI
Esnaola et al^18^	2002	245	OLT	Tumor size greater than 4 cm (OR, 3.0; 95% CI, 1.2–7.1, *P* < 0.01) High tumor grade (OR, 6.3; 95% CI, 2.0–19.9, *P* < 0.01)
Eguchi et al^19^	2010	229	Resection or OLT	Tumor size (OR, 1.6; 95% CI, 1.3–2.2, *P* < 0.01) High tumor grade (OR, 60.2; 95% CI, 2.5–1408, *P* < 0.05) Level of DCP (*P* < 0.001) Level of AFP (*P* < 0.05)
Kim et al^20^	2008	190	Resection	Tumor size of > 5 cm (RR = 2.9; 95% CI, 2.138–9.491, *P* = 0.043) Tumor numbers of > 3 (RR = 4.241; 95% CI, 2.569–9.343, *P* = 0.011) Histologic grades Edmondson grades 3–4 (RR = 45.110; 95% CI, 6.596–31.242, *P* = 0.001)
Chou et al^33^	2014	102	Resection or OLT	Tumor size (OR, 1.088; 95% CI, 0.811–1.460, *P* = 0.572) Histologic grade (OR, 1.728; 95% CI, 0.543–5.494, *P* = 0.354) Radiologic evidence of tumor capsule (OR, 3.250; 95% CI, 0.932–11.327, *P* = 0.064)
Lei et al^24^	2016	1004	Resection	Tumor margin (OR, 28.828; 95% CI, 7.718–107.680, *P* < 0.001) HBV DNA load of > 10^4^ (OR, 2.31; 95% CI, 1.66–3.20, *P* < 0.001) AFP of > 400 ng/ml (OR, 3.32; 95% CI, 2.25–4.91, *P* < 0.001) Tumor diameter (OR, 1.54; 95% CI, 1.32–1.79, *P* < 0.001) Multiple tumors (OR, 3.03; 95% CI, 1.67–5.48, *P* < 0.001) Incomplete tumor capsule (OR, 3.15; 95% CI, 2.24–4.42, *P* < 0.001) Peripheral tumor location (OR, 1.48; 95% CI, 0.96 to 2.29, *P* < 0.07) Non-smooth tumor boundary (OR, 0.84; 95% CI, 0.54–1.29, *P* < 0.42)
Ariizumi et al^36^	2011	135	Resection	Incomplete radiological capsule in the dynamic images (OR, 1.538; 95% CI, 0.231–10.228; *P* = 0.656) Non-smooth Tumor margin (OR, 18.814; 95% CI, 1.467–241.343; *P* = 0.024)
Poté et al^78^	2015	128	Resection or OLT	PIVKA-II level > 90 mAU/ml (HR, 3.5; 95% CI, 1.08–11.8, *P* = 0.043) Moderately/poorly differentiated tumor (HR, 3.4; 95% CI, 1.04–11.05, *P* = 0.037)
Li et al^28^	2019	469	Resection	HBV DNA level > 2000 (HR, 1.421; 95% CI, 1.018–1.984, *P* < 0.001) Incomplete tumor encapsulation (HR, 1.697; 95% CI, 1.294–2.225, *P* < 0.001) Invasion of the liver capsule (HR, 1.498; 95% CI, 1.142–1.964, *P* = 0.004)
Xu et al^77^	2016	108	Resection	Tumor ciRS-7 expression (OR, 4.08; 95% CI, 1.06–15.74, *P* = 0.041) GVI (OR, 12.89; 95% CI, 1.85–89.63, *P* < 0.01) AFP of > 400 ng/ml (OR, 7.68; 95% CI, 2.33–25.26, *P* = 0.001) Liver cirrhosis (OR, 5.86; 95% CI, 1.65–20.84, *P* = 0.006)

*MVI* microvascular invasion, *AFP* α-Fetoprotein, *OLT* orthotopic liver transplantation, *OR* odds ratio, *CI* confidence interval, *RR* relative risk, *DCP* des-γ-carboxy prothrombin, *Gd-EOB-DTPA* gadolinium-ethoxybenzyl diethylenetriamine pentaacetic acid, *MRI* magnetic resonance imaging, *HR* hazard ratio, *PIVKA-II* Prothrombin induced by vitamin K absence-II, *GVI* gross vascular invasion defined as the tumor embolus were observed in the first or second branches of the portal veins by preoperative radiological tests, such as CT or MRI, *ciRS-7* Circular RNA sponge for microRNA-7, *OS* overall Survival.

According to the classification criteria of Eggel, HCC can be roughly classified into four categories: type I, single nodular (SN) type; type II, single nodular type with extranodular growth (SNEG); type III, confluent multinodular type (CMN); and type IV, infiltrative type^13^. According to previous studies, the incidence of type I fluctuates between 7.7% and 45.8%, which is lower than the incidences of type II (25–93.4%) and type III (12.5 ~ 100%) MVI^14,15^. He et al. found that the incidence of type I, type II and type III MVI were 36%, 32.4% and 30.3%, respectively, while the incidence of type IV MVI was 63.5%, which was substantially higher than the incidences of the first three types^16^. The above data indicate that the incidence of MVI may be correlated with the general classification of HCC.

### Tumor diameter

Tumor diameter is correlated with the incidence of MVI because the larger the liver tumor diameter is, the higher the incidence of MVI and intrahepatic metastasis, which often predicts a poor prognosis^17^. A cohort of 245 patients who underwent resection for HCC were enrolled in a clinical study. Twenty-five percent of patients with tumors smaller than < 2 cm had MVI, whereas 31% and 50% of patients with tumors 2–4 cm in size or larger than 4 cm, respectively, had MVI^18^. Eguchi et al. found that 127 of 229 patients with HCC who underwent curative liver resection had HCC without microscopic portal vein invasion, and 52 had HCC with microscopic portal vein invasion. Among them, the mean diameters of the tumors in the MVI positive group and the MVI negative group were 5.2 cm and 3.0 cm, respectively^19^.

### Degree of tumor differentiation

According to the Edmondson-Steiner pathological classification system, tumor differentiation can be divided into the high differentiation type (I), medium differentiation type (II) and low differentiation type (III–IV)^20^. There are relationships between the invaded vasculature and MVI and the pathological grade of the tumor. Regarding the association of vascular invasion with histologic grade, when stratified by tumor stage, patients with Edmondson grade I and tumor size < 5 cm had no MVI, while those with Edmondson grade II or higher had relatively higher incidences of MVI, even if they had small tumors (< 2 cm)^21^. Furthermore, previous research results have shown that epithelial to mesenchymal transition (EMT)-related genes, including SNAIL, FoxC1, and vimentin, are upregulated in HCC with MVI, implying that EMT is associated with the development of MVI^22^. Zhang et al. demonstrated the important prognostic value of the FoxM1-KIAA0101 axis in HCC patients and highlighted the essential role of KIAA0101 in FoxM1-driven HCC invasion and metastasis based on the regulation of genes involved in EMT^23^.

### Hepatitis B virus (HBV) and steroid hormone

HBV is associated with 70–90% of HCC cases, and more than 70% of all newly diagnosed HCC cases occur in highly endemic areas for HBV in the Asia–Pacific region, particularly in China^24,25^. Chronic HBV infection is a major risk factor for the development of liver cirrhosis and HCC and is also associated with an increased recurrence rate and a decreased survival rate of HCC after surgical resection^26^. Previous studies have confirmed that the HBV-initiated tumorigenic process may play a role in the development of MVI in HCC^27,28^. Lei et al. established a novel prediction model for the preoperative prediction of the presence of MVI in HBV-related HCC, in which a high HBV DNA load (> 10^4^ IU/ml) was independently associated with the development of MVI^24^. The results from Wei et al. also showed that compared with patients without HBV infection, HBV-infected patients with HCC had a significantly higher incidence of MVI^29^.

As a sexually dimorphic organ, the liver can express the estrogen receptors (ERs) ERα and ERβ, regulating the expression of various genes involved in the cell cycle, proliferation, apoptosis and inflammation^30^. Patients could be protected from HCC by estrogen, which is considered to be a preventive factor, and achieve better recovery after HCC treatment^31^. The findings of one study indicated that males express significantly higher levels of ERα than females but comparable levels of ERβ. The ERα: ERβ expression ratio in males is also significantly higher than that in females^32^. However, whether these differences in ER subtype expression predispose males to rapid HCC disease progression needs further investigation.

### Tumor capsule

The integrity of the tumor envelope is associated with MVI, and the risk of MVI also increases with the decrease in the integrity of the tumor envelope. The tumor envelope is affected by distensible tumor growth, compressing the surrounding normal liver tissue and allowing fibrous tissue to proliferate and form around the tumor. Most scholars believe that the integrity of the tumor envelope can prevent contact between tumor cells and surrounding tissues and limit the exogenous invasive growth of tumors, while an incomplete or absent envelope can result in the tumor being more likely to invade the surrounding tissues. Chou et al. analyzed the preoperative CT data of 102 patients with HCC including tumor size, tumor capsule, tumor margins and peritumoral enhancement, and the postoperative pathological results^33^. They found that nonsmooth margins (presenting as the focal outgrowth of nodules protruding into the nontumor parenchyma) and nonsmooth margins with the multinodular type were associated with MVI. They thus thought that nonsmooth margins detected on multiphasic CT may be predictive of MVI in HCC. Renzulli et al^34^ have indicated that peritumoral enhancement is a significant marker of histologic MVI and that radiographic tumor margins may be radiological indicators for predicting MVI in patients with HCC. It is possible to assess peritumoral enhancement by using noninvasive techniques, such as CT or dynamic MR imaging, which are recommended for diagnosing HCC. In particular the newly introduced MRI contrast agent gadoxetic acid (gadolinium-ethoxybenzyl-diethylenetriamine pentaacetic acid, Gd-EOB-DTPA) has enabled the concurrent assessment of tumor vascularity and hepatocyte-specific contrast enhancement during the hepatobiliary phase (HBP), which can help to detect and characterize smaller HCCs and their precursors^35,36^. The organic anion transporter peptides (OATP) 1B1 and 1B3 are liver-specific molecules, and they are transporters of cholephilic organics, playing an important role in the uptake of the MRI contrast media Gd-EOB-DTPA. Therefore, OATP, identified by this unique technique (MRI) in HCC, could be used together with K7/19 to identify a phenotypical spectrum of HCC progression^37^. In summary, whether it CT or MRI is used, there are significant associations between MVI and peritumoral enhancement and peritumoral hypointensity^38^.

Although a number of clinical features, including tumor size, capsule integrity, pathological grade, and imaging manifestations are correlated with the occurrence of MVI, the accurate prediction of MVI cannot be evaluated by a single indicator^39^. Instead, a comprehensive analysis of multiple factors is needed for a valuable clinical prediction scheme. A study conducted in China, that included 1004 samples of HBV-related HCC found that preoperative factors, including multiple tumors, a large tumor diameter, an incomplete tumor capsule, a high serum α-fetoprotein level, an HBV DNA load greater than 10^4^ IU/ml, a platelet count less than 100 × 10^3^/µl, and the presence of a typical dynamic pattern on contrast-enhanced MRI, were significantly associated with MVI^24^. Based on the above seven indicators, they constructed a prediction model that performed well and was supported by C-index values of 0.81 and 0.80 in the training and validation cohorts, respectively. The optimal calibration curves demonstrated good agreement between the predictions and actual observations. As a pathological indicator, MVI is often difficult to measure accurately before surgery. Therefore, researchers have attempted to explore methods of preoperatively predicting MVI in a large number of studies to enable the more accurate evaluation of tumor invasion, and the formulation of comprehensive targeted treatment plans during and after surgery.

## Clinical significance of MVI

### Preoperative prediction of MVI guides surgical planning (Table 2)

**Table 2:** Summary of the ongoing clinical studies in HCC with MVI.

Author	Year	N	Treatment	MVI	Results
Shi et al^42^	2019	231	AR (118) versus NAR (113)	MVI positive group (113)	AR was superior to LR in both RFS and OS when MVI was positive. (*P* = 0.001 and 0.001 for RFS and *P* = 0.009 and 0.002 for OS) Wide margins was superior to narrow margins in both RFS and OS when MVI was positive.(*P* = 0.006 and 0.010)
Liu et al^40^	2018	84	AR (35) versus NAR (49)	All patients were MVI positive	The 1-year, 2-year and 3-year progression-free survival rate were 80.0%, 62.9%, 51.4% in the AR group and 71.4%, 49.0%, 38.8%, in the NAR group, respectively The 1-year, 2-year and 3-year overall survival rate were 85.7%, 68.6%, 57.1% in the AR group and 79.6%, 53.1%, 42.9% in the NAR group, respectively
Wei et al^60^	2018	250	Curative resection	All patients were MVI positive	The median DFS was longer in the PA-TACE group than in the hepatectomy alone group [17.45 months (95% CI, 11.99–29.14) versus . 9.27 months (95% CI, 6.05–13.70), HR = 0.70 (95% CI, 0.52–0.95), *P* = 0.020] The median OS was also longer in the PA-TACE group than in the hepatectomy alone group [44.29 months (95% CI 25.99–62.58) versus . 22.37 months (95% CI 10.84–33.91), HR = 0.68 (95% CI 0.48–0.97), *P* = 0.029]
Wang et al^51^	2019	1630	Curative resection	All patients were MVI positive	The OS and DFS rates were significantly different between the postoperative adjuvant TACE group and the operation only group (HR 0.57; 95%CI, 0.48 ~ 0.68, *P* < 0.00001; HR 0.66; 95%CI, 0.58 ~ 0.74, *P* < 0.00001)
Peng et al^76^	2019	260	Curative hepatectomy	MVI positive (127) and MVI negative (133)	For patients with MVI-positive lesions, the median OS and PFS after combination treatment were longer than those after TACE alone (OS: 17.2 months vs. 12.1 months, *P* = 0 .02; PFS: 17.0 months vs. 11.0 months, *P* = 0.02)
Wang et al^63^	2018	271	Curative hepatic resection	MVI positive (128) and MVI negative (143)	Both DFS and OS were longer in the PA-TACE group than in the hepatic resection alone group (5-year DFS, 26.3% vs. 20.7%, *P* = 0.038; 5-year OS, 73.6% vs. 47.7%, *P* = 0.005) No differences were noted in DFS and OS among MVI negative patients with or without PA-TACE (5-year DFS, 33.7% vs. 33.0%, *P* = 0.471; 5-year OS, 84.1% vs. 80.3%, *P* = 0.523)
Chen et al^52^	2019	2190	Curative resection	All patients were MVI positive	Adjuvant TACE showed better 1-, 3-, and 5-OS rates compared to LR alone. (OR = 0.33, *P* < 0.001; OR = 0.49, *P* < 0.001; and OR = 0.59, *P* < 0.01) Adjuvant TACE showed better 1-, 3-, and 5-DFS compared to LR alone. (OR = 0.45, *P* < 0.001; OR = 0.50, *P* < 0.001; and OR = 0.58, *P* < 0.001)
Huang et al^65^	2019	49	Curative resection	All patients were MVI positive	The RFS and OS were both longer in the sorafenib group, and the 3-years RFS rates of the sorafenib group and control group were 56.3% and 24.2% (*P* = 0.027). And the 3-years OS rates of the sorafenib group and control group were 81.3% and 39.4% (*P* = 0.006)
Zhang et al^75^	2019	728	R0 Curative resection	All patients were MVI positive	OS was significantly better for patients in the PA-sorafenib group than the LR group (1 year, 65% vs. 85%; 3 years, 51% vs. 66%; 5 years, 37% vs. 57%; *P* = 0.007,). RFS was significantly better for patients in the PA-sorafenib group than the LR group (1 year, 55% vs. 72%; 3 years, 36% vs. 47%; 5 years, 19% vs. 39%; *P* = 0.001)
Sun et al^59^	2015	322	R0 hepatectomy	All patients were MVI positive	The 1-, 2-, 3-, and 5-year RFS rates were respectively 69.3, 55.5, 46.7, and 35.0% for the PA-TACE group and 47.0, 36.2, 34.1, and 30.3% for the RH group (*P* = 0.012) The 1-, 2-, 3-, and 5-year OS rates were respectively 94.2, 78.8, 71.5, and 54.0% for the PA-TACE group and 78.9, 62.2, 54.1, and 43.2% for the RH group (*P* = 0.006)

*MVI* microvascular invasion, *TACE* transcatheter arterial chemoembolization, *PA* postoperative adjuvant, *RFS* recurrence free survival, *AR* anatomic resection, *NAR* non-anatomic resection, *OR* odds ratio, *CI* confidence interval, *HR* hazard ratio, *LR* liver resection, *RR* re-resection, *RFA* radiofrequency ablation, *RT* ratio therapy, *RH* R0 hepatectomy.

For malignant tumors, adequate surgical margins are guaranteed for radical surgery. However, for liver resection, due to the particularity of the tumor location or degree of liver sclerosis, the scope of surgical resection is often limited, so it is sometimes difficult to ensure adequate surgical margins^40^. In addition, the width of the surgical resection margin has always been controversial, especially in patients with cirrhosis. Some scholars believe that excessive resection of normal liver tissue may not affect the prognosis of patients but may increase the incidence of postoperative complications. Some scholars argue that anatomic resection of HCC can simultaneously remove both the lesion and micrometastases that may have invaded the portal vein or flowed along the branch of the portal vein into the liver segment beyond the main tumor. If this is true, the anatomic resection of HCC could effectively guarantee an adequate scope of resection and theoretically reduce the postoperative recurrence due to micrometastases in the residual tissues that is observed after nonanatomic resection^41,42^. A systematic review and meta-analysis from Moris et al^41^ indicated no difference in perioperative complications or perioperative mortality but a higher level of blood loss among patients undergoing anatomic resection versus those undergoing nonanatomic resection; these findings were due to the fact that the resection margin was slightly wider following anatomic resection than after nonanatomic resection. Anatomic resection was associated with an obvious disease-free survival (DFS) benefits at 1-, 3- and 5 years. In addition, anatomic resection seemed to offer an advantage over nonanatomic resection in terms of DFS and overall survival (OS) among patients undergoing resection of HCC, especially among those without cirrhosis. Portolani N et al. also found that for patients undergoing nonanatomic resection, the early recurrence ratio could reach as high as 76.2% in the first 24-month period^43^. Therefore, multiple meta-analyses and current mainstream research suggest that anatomic resection appears to benefit the DFS and OS of patients with HCC, especially those without cirrhosis^41,44^. MVI is not only an important predictor of the risk of the postoperative recurrence of liver cancer but also an important pathological indication of the efficacy of antitumor therapy of clinical HCC after an operation. According to clinical data presented by Feng et al^7^, 41.9% of patients with HCC were pathologically positive for MVI, and the surrounding area near the tumor (< 1 cm) was found to be a high-incidence area for MVI. Studies^45^ have also found that compared with the M1 and M0 groups, the M2 group had significantly lower OS and DFS rates. In addition, the liver resection method and tumor diameter of the M2 group were independent risk factors for OS and DFS after hepatectomy. Therefore, caution should be exered when considering the choice of surgical methods for liver cancer patients with MVI. As mentioned above, although a number of studies have used individual factors to predict preoperative MVI, including tumor size, capsule integrity, CT/MRI tumor characteristics, or multiple indicators to establish evaluation models, it is still difficult to accurately predict the occurrence of MVI before surgery at this time. A tumor diameter greater than 2 cm, multiple tumors, an incomplete tumor capsule, the presence of a typical dynamic pattern and a high serum α-fetoprotein (AFP) level are independently associated with MVI. Therefore, for patients with these characteristics, anatomic resection may be a relatively better option if their liver function is preoperatively assessed as being adequate and sufficient remaining liver tissue will exist. If the hepatic functional reserve of the patient is poor or the remaining liver volume is insufficient, nonanatomical resection can be selected, but the surgical margin must be greater than 1 cm. Only in this manner can the liver function of the patient be guaranteed and the risk of early local recurrence be reduced.

### Prophylactic treatment with transarterial chemoembolization (TACE) after liver resection

The mechanism of underlying early recurrence after resection of HCC is very complex. The postoperative recurrence of HCC is primarily driven by intrahepatic diffusion and is most common near the surgical resection margin; this finding may be related to a compensatory increase in blood supply or incomplete surgical resection resulting in residual MVI^46^. TACE, which is used to treat intermediate-stage HCC, is currently recognized as one of the most commonly used nonsurgical HCC treatment methods^47,48^. TACE can decrease blood flow to the tumor, and ischemic tumor necrosis can be induced via the arterial injection of chemotherapeutic drugs and embolizing agents^49^. The effectiveness of TACE as an adjuvant therapy in HCC has been documented in clinical studies^50–52^; however, whether all HCC patients need routine prophylactic use of TACE after surgery remains controversial. Because TACE may reduce remnant liver function and immune function and because recurring tumors usually have different clonal origins from primary tumors^53,54^, Jiang JH et al. believed that postoperative adjuvant TACE does not improve OS or reduce recurrence in HCC patients^55^. However, that study was a retrospective analysis that was likely subject to subtle selection biases, even after propensity score matching. Nevertheless, a randomized controlled trial (RCT) by Li JQ et al. showed postoperative adjuvant TACE to be beneficial for patients with HCC lesions larger than 5 cm in diameter, multiple nodules, macroscopic vascular invasion^56^ or macroscopic portal vein tumor thrombus^57^. Another study^58^ reached a similar conclusion: adjuvant TACE (two or three cycles) after radical resection is beneficial for HCC patients with poor differentiation and MVI, especially for those with a tumor diameter > 5 cm. Similarly, postoperative adjuvant TACE significantly increased RFS and OS and reduced the early recurrence rate of HCC patients with MVI, and postoperative adjuvant TACE was found to be an independent risk factor for postoperative RFS and OS^59^. The significant effect of postoperative adjuvant TACE on HCC patients with high risk factors for recurrence may be attributed to the following reasons^60^. Tumor cells in HCC patients with MVI tend to form micrometastases that travel through the blood system to normal tissues surrounding the tumor, resulting in poor DFS and OS^61,62^. Postoperative adjuvant TACE after liver resection could kill tumor cells and improve the survival rate of patients^63^. Furthermore, micrometastatic lesions could also be addressed in this way^64^. In conclusion, for liver cancer patients with high-risk recurrence factors, especially those with MVI, postoperative adjuvant TACE treatment has gradually become the recommended treatment method.

### Future of HCC treatment with molecular targeted therapy and immunotherapy

Over the past decade, the molecular-targeted drug sorafenib has been introduced and has been shown to inhibit tumor growth through multiple mechanisms. Sorafenib^65^ is a multitargeted, orally active small molecule tyrosine kinase inhibitor (TKI) that inhibits Raf kinase and the vascular endothelial growth factor receptor (VEGFR) intracellular kinase pathway; this drug is promising for the treatment of patients with advanced HCC^66^. The multicenter European SHARP trial^67^ and Asian-Pacific trial^68^ established sorafenib monotherapy as the new reference standard systemic treatment for advanced HCC and formed the basis for the approval of sorafenib for treatment of unresectable HCC. Lenvatinib is an inhibitor of VEGFR-1, VEGFR-2, and VEGFR-3, as well as fibroblast growth factor receptors (FGFRs), platelet-derived growth factor receptor (PDGFR) alpha, RET, and KIT. In the REFLECT study^69^, lenvatinib was compared with sorafenib in 954 patients with unresectable HCC, and it was considered noninferior to sorafenib. Based on the REFLECT trial, lenvatinib received approval for the first-line treatment of unresectable HCC in 2018. A benefit associated with regorafenib in patients progressing after first-line treatment with sorafenib was suggested in the RESORCE trial^70^. Regorafenib is also promising in patients with oral drug resistance to sorafenib and has become the first approved second-line treatment for HCC. Checkpoint inhibitor immunotherapy is an important research direction in generalized immunotherapy. Nivolumab and pembrolizumab are human monoclonal antibodies that target the programmed cell death 1 receptor (PD-1), restoring T cell immune activity directed against tumor cells. The median OS times in advanced HCC patients given nivolumab and perbrolizumab were 15.6 months and 12.9 months, respectively, and they are currently approved for second-line HCC treatment^71,72^.

Many studies have shown significantly better survival outcomes by combining sorafenib with radiofrequency ablation or TACE to treat HCC^73,74^. However, due to the high degree of biological heterogeneity of HCC, the benefits of adjuvant sorafenib in the subgroup of HCC patients with MVI need to be further explored. A total of 728 HCC patients with MVI in the resected specimens after R0 resection were enrolled in a propensity score matching analysis^75^. In that study, the OS and RFS in HCC patients with MVI who underwent R0 resection and were given postoperative adjuvant therapy with sorafenib were significantly better than those in the patients who underwent liver resection alone. This indicates that patients with MVI after R0 resection can benefit from postoperative adjuvant sorafenib. Peng et al^76^ also found that the combination of TACE and sorafenib might play an important role in the improvement in the survival of patients with MVI-positive lesions. The above conclusions also indicate that patients with MVI after the resection of HCC may benefit from postoperative adjuvant oral sorafenib combination therapy. Multicenter studies need to be conducted. However, regarding lenvatinib, regorafenib and immunotherapy checkpoint inhibitors, their clinical applications are still supported by data from clinical trials due to their short clinical application time.

## Future directions

MVI is an important pathological predictor of the risk of the postoperative recurrence of HCC. MVI is an important factor guiding surgical planning and the formulation of comprehensive postoperative treatment plans. We can make preoperative predictions based on the AFP level, des-gamma carboxyprothrombin (DCP) level, tumor size, circular RNA^77^, imaging characteristics and PIVKAII^78^ or postoperative MVI determined through a postoperative histological examination. The preoperative prediction of MVI can reduce the risk of postoperative recurrence by indicating the use of antiviral drugs in advance and the need for the expansion of the scope of surgical resection. Postoperative adjuvant TACE combined with sorafenib could improve DFS and OS in HCC patients with MVI. However, the correlation between MVI and prognosis also has certain limitations in HCC. In addition to MVI, there are a series of interfering factors affecting the prognosis of patients with intermediate-advanced HCC, such as the presence of satellite lesions, a heavy tumor burden and persistent HBV replication. Similarly, the presence of MVI can also be detected in early-advanced stage HCC patients with good liver function; however, the prognostic importance of MVI is not strong at this stage of HCC^79^. Traditionally speaking, when stratified by BCLC stages, MVI is a powerful predictor of outcomes in BCLC HCC stages A and B. However, after propensity score matching (PSM) analysis, MVI was found to independently predict OS in BCLC stage A HCC and not BCLC stage B^80^. Therefore, in MVI-positive HCC patients, the choice of postoperative therapy, such as TACE and targeted therapy, and BCLC stage are also important reference factors.

Although there are various ways to treat HCC, each treatment has its own advantages and limitations. More importantly, when determining the appropriate therapy for HCC, we must consider both tumor-related factors and background liver-related factors. For BCLC stage A HCC patients with MVI positivity, anatomic resection is recommended based on the prediction model. For these patients, postoperative adjuvant TACE combined with sorafenib is also a very important treatment. Liver resection and TACE are recommended for BCLC intermediate stage HCC patients without MVI and with MVI, respectively^81^. For HCC patients with MVI, we can formulate more individualized programs, such as shortening the review time, to reduce as much as possible in clinical practice the early recurrence rate caused by high-risk factors.

Worldwide, in the past 20 years, although the research field of HCC has made remarkable progress, there are still many difficulties worth studying and solving. Surgical treatment is still the preferred treatment for HCC. For HCC patients with MVI, the preoperative prediction of MVI may play a certain guiding role in planning procedures, and the comprehensive sequential postoperative pathological detection of HCC MVI may provide a basis for treatment decisions. The methods of combining molecular targeted therapies and immunotherapies in personalized diagnosis and treatment plans for patients while at the same time reducing the side effects of drug therapy will become be the focus of future HCC research.

## Abbreviations

HCC: Hepatocellular carcinoma
TACE: Transarterial chemoembolization
TKI: Tyrosine-kinase inhibitors
MVI: Microvascular invasion
HBV: Hepatitis B virus
HCV: Hepatitis C virus
OS: Overall survival
RFS: Relapse free survival
